# Quantitative assessment of the central versus peripheral effect of intravenous clonidine using baroreflex equilibrium diagrams

**DOI:** 10.1186/s12576-021-00824-y

**Published:** 2021-12-31

**Authors:** Toru Kawada, Takuya Nishikawa, Yohsuke Hayama, Meihua Li, Can Zheng, Kazunori Uemura, Keita Saku, Tadayoshi Miyamoto, Masaru Sugimachi

**Affiliations:** 1 Department of Cardiovascular Dynamics, National Cerebral and Cardiovascular Center, Osaka, 564-8565 Japan; 2 Department of Sport and Health Sciences, Faculty of Sport and Health Sciences, Osaka Sangyo University, Osaka, 559-0034 Japan

**Keywords:** Clonidine, Carotid sinus baroreflex, Open-loop systems analysis, Baroreflex equilibrium diagram, Rats

## Abstract

Clonidine is a first-generation central antihypertensive that reduces sympathetic nerve activity (SNA). Although clonidine also exerts peripheral vasoconstriction, the extent to which this vasoconstriction offsets the centrally mediated arterial pressure (AP)-lowering effect remains unknown. In anesthetized rats (*n* = 8), we examined SNA and AP responses to stepwise changes in carotid sinus pressure under control conditions and after intravenous low-dose (2 μg/kg) and high-dose clonidine (5 μg/kg). In the baroreflex equilibrium diagram analysis, the operating-point AP under the control condition was 115.2 (108.5–127.7) mmHg [median (25th–75th percentile range)]. While the operating-point AP after low-dose clonidine was not significantly different with or without the peripheral effect, the operating-point AP after high-dose clonidine was higher with the peripheral effect than without [81.3 (76.2–98.2) mmHg vs. 70.7 (57.7–96.9), *P* < 0.05]. The vasoconstrictive effect of clonidine partly offset the centrally mediated AP-lowering effect after high-dose administration.

## Introduction

Clonidine is a first-generation antihypertensive agent that suppresses sympathetic outflow from the central nervous system by acting on α_2_-adrenergic and I_1_-imidazoline receptors [1–3]. After clonidine, several central antihypertensive agents with different properties have been developed. Rilmenidine and moxonidine preferentially act on I_1_-imidazoline receptors [1–4], thereby reducing side effects associated with central α_2_-adrenergic stimulation, such as dry mouth and sedation. Guanfacine is selective for α_2A_, a subtype mainly responsible for central sympathetic suppression [5].

Despite classified as a central antihypertensive agent, the effect of a given drug on arterial pressure (AP) is not determined by its central action alone when the drug is administered systemically. In our previous study, intravenously administered moxonidine increased AP at any given sympathetic nerve activity (SNA), suggesting that a peripheral effect of moxonidine significantly offset the centrally mediated AP-lowering effect [6]. Intravenously administered guanfacine also exhibited a peripheral effect that nearly canceled the reduction of AP at the operating point of the arterial baroreflex [7]. By contrast, intravenously administered rilmenidine showed a weak vasoconstrictive effect that was unmasked by opening the baroreflex negative feedback loop [8].

Regarding clonidine, how exactly the central and peripheral effects interact to determine AP remains unknown. Since clonidine may serve as a reference drug to evaluate other central antihypertensive agents, we quantified the effect of clonidine on the baroreflex-mediated sympathetic AP regulation in the present study. Intravenously administered clonidine typically induces an initial AP elevation through peripheral vasoconstriction but eventually decreases AP through central sympathetic suppression. We hypothesized that the peripheral vasoconstrictive effect of clonidine persists even during the hypotensive phase and partly offsets the centrally mediated AP-lowering effect.

## Materials and methods

### Surgical preparation

Animal care was provided in strict accordance with the Guiding Principles for the Care and Use of Animals in the Field of Physiological Sciences, which has been approved by the Physiological Society of Japan. The experimental protocols were reviewed and approved by the Animal Subject Committee of the National Cerebral and Cardiovascular Center (No. 20008, 21009).

Eight male Wistar-Kyoto rats (304–395 g) were anesthetized with an intraperitoneal injection (2 mL/kg) of a mixture of urethane (250 mg/mL) and α-chloralose (40 mg/mL). The anesthetic mixture was diluted 18-fold and administered continuously (2 mL·kg^−1^ h^−1^) from the right femoral vein. The rats were mechanically ventilated with oxygen-supplied air. AP was measured from the right femoral artery, and the heart rate (HR) was detected from a body surface electrocardiogram. A pair of stainless-steel wire electrodes (AS633, Cooner Wire, CA, USA) was attached to a postganglionic branch of the left splanchnic sympathetic nerve and fixed with silicone glue (Kwik-Sil, World Precision Instruments, FL, USA). The nerve activity was amplified using a biosignal amplifier (AB-610J, Nihon Kohden, Japan) with a bandpass filter setting between 150 and 1000 Hz. The amplified signal was then full-wave rectified and low-pass filtered at a cut-off frequency of 30 Hz to quantify SNA. Bilateral carotid sinus baroreceptor regions were isolated from the systemic circulation [9, 10], and the carotid sinus pressure (CSP) was controlled with a servo-pump system (ET-126, Labworks, Costa Mesa, CA, USA). Bilateral vagal and aortic depressor nerves were sectioned at the neck to minimize reflex effects from the cardiopulmonary region and aortic arch.

### Protocol

After completing the surgical preparation, we waited for at least 30 min to obtain stable hemodynamics. During the waiting period, CSP was servo-controlled to mimic instantaneous AP. Although pressure waveforms did not exactly match between the CSP and AP signals due to the limited performance of the servo controller, the baroreflex negative feedback loop was effectively closed.

**Fig. 1:**
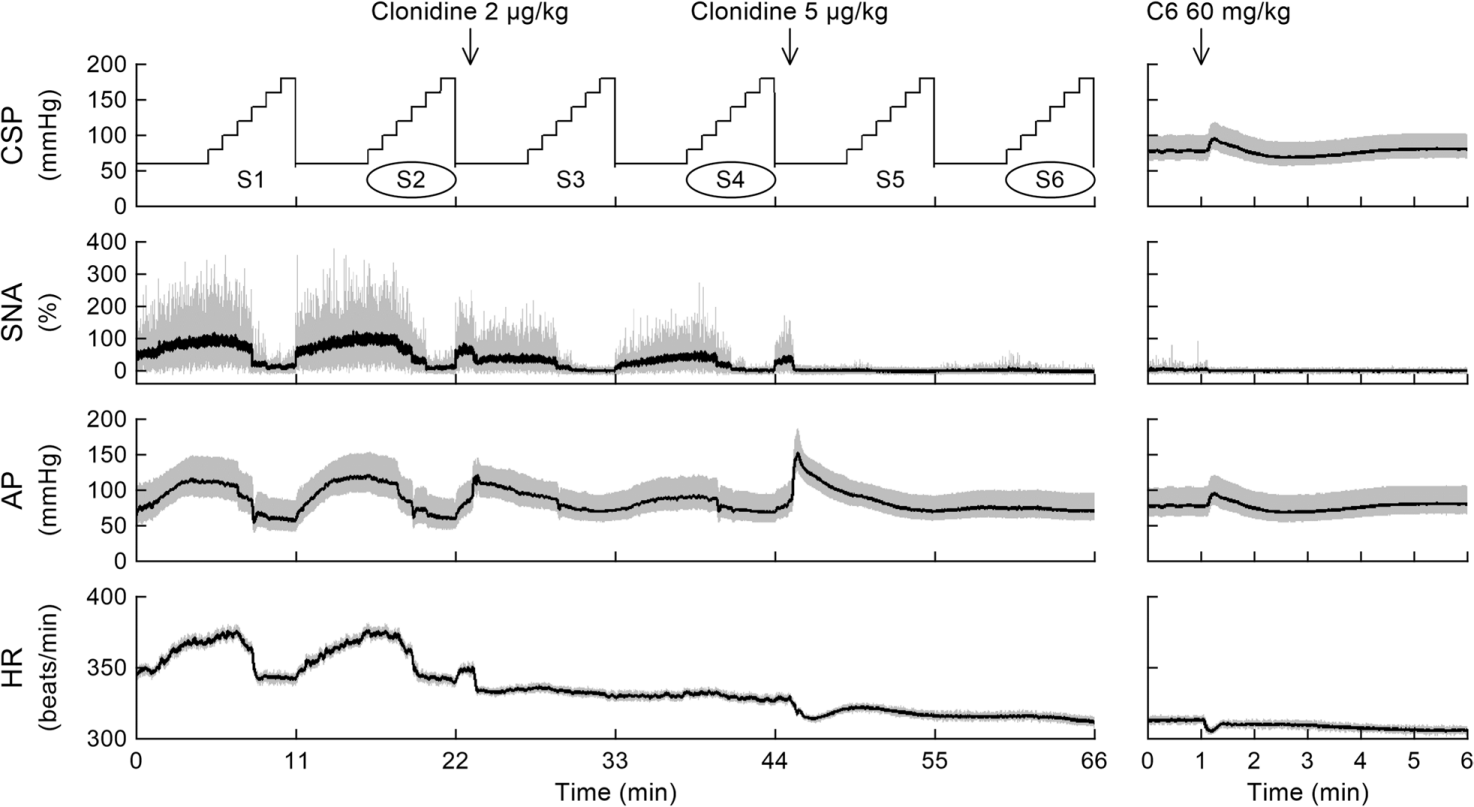
Time series of carotid sinus pressure (CSP), sympathetic nerve activity (SNA), arterial pressure (AP), and heart rate (HR) obtained in one rat. In the left panels, stepwise CSP changes were repeated and referred to as S1 to S6. One minute after the completion of S2, low-dose (2 μg/kg) clonidine was intravenously administered. One minute after the completion of S4, high-dose (5 μg/kg) clonidine was intravenously administered. The baroreflex response in S2 was treated as the control, whereas the responses in S4 and S6 were used to evaluate the effects of low-dose and high-dose clonidine, respectively. In the right panels, hexamethonium bromide (C6) was intravenously administered. CSP was servo-controlled to mimic instantaneous AP during the C6 protocol. The CSP and AP signals are virtually the same at this time resolution. SNA was depicted as a 10-Hz resampled signal (gray) and a 2-s moving averaged signal (black). AP and HR were depicted as 200-Hz resampled signals (gray) and 2-s moving averaged signals (black)

To estimate the open-loop static characteristics of the carotid sinus baroreflex, CSP was first decreased to 60 mmHg for 5 min, and then increased stepwise to 180 mmHg in increments of 20 mmHg every minute. The stepwise CSP input was repeated, and the sequences were referred to as S1 to S6 (Fig. 1, left panels). One minute after the completion of S2, low-dose (2 μg/kg) clonidine (clonidine hydrochloride, Tokyo Chemical Industry; 2 μg/mL dissolved in physiological saline) was administered from the left femoral vein. One minute after the completion of S4, high-dose (5 μg/kg) clonidine (5 μg/mL dissolved in physiological saline) was added. The effect of high-dose clonidine may need to be interpreted as a cumulative one.

At the end of the protocol, CSP was again servo-controlled to mimic instantaneous AP. Under the baroreflex closed-loop condition, hexamethonium bromide, a ganglionic blocker, was intravenously administered (60 mg/kg), and the noise level of SNA was assessed. Approximately 3 min after the injection of hexamethonium bromide, mean AP was obtained as a 10-s averaged value.

### Data analysis

Data were recorded on a laboratory computer system using a 16-bit analog-to-digital converter. We treated the baroreflex response in S2 as a control and evaluated the effects of low-dose and high-dose clonidine from the responses in S4 and S6, respectively. In each of S2, S4, and S6, the SNA, AP, and HR data were averaged for the last 10 s at each CSP level. As the absolute amplitude of SNA varied among animals depending on the recording conditions, SNA was normalized in each animal based on the value at the CSP of 60 mmHg in S2 (100%) and the value after ganglionic blockade (0%). The relationships between CSP and AP (the total reflex arc), between CSP and HR, and between CSP and SNA (the neural arc) were analyzed using a four-parameter logistic function as follows [11, 12]:

$$y = \frac{{P_{1} }}{{1 + \exp \left[ {P_{2} \left( {{\text{CSP}} - P_{3} } \right)} \right]}} + P_{4} ,$$

where *y* denotes the output variable, and *P*_1_, *P*_2_, *P*_3_, and *P*_4_ represent the response range, slope coefficient, midpoint pressure on the CSP axis, and the lower asymptote of the sigmoid curve, respectively.

The relationship between SNA and AP (the peripheral arc) was quantified using linear regression analysis [12]:

$${\text{AP}} = b_{0} + b_{1} \times {\text{SNA,}}$$

where *b*_0_ and *b*_1_ represent the intercept and slope of the regression line, respectively.

The operating point of the carotid sinus baroreflex was determined from the intersection point between the fitted neural and peripheral arcs on a baroreflex equilibrium diagram [12–14]. The operating-point AP values were estimated under conditions of control, after low-dose clonidine, and after high-dose clonidine. To exclude the peripheral effect of clonidine, the intersection points after clonidine were also estimated by reflecting changes in the neural arc alone.

### Statistical analysis

Data are presented as box and whisker plots except otherwise specified. The fitted parameter values of the baroreflex static characteristics were compared among conditions of control, after low-dose clonidine, and after high-dose clonidine using one-way repeated-measures analysis of variance (ANOVA) followed by a Tukey test. The Geisser-Greenhouse's correction was applied (Prism 8, GraphPad Software, San Diego, CA, USA). The operating-point AP values estimated under conditions of control, after low-dose clonidine (with or without the peripheral effect), and after high-dose clonidine (with or without the peripheral effect) were compared using one-way repeated-measures ANOVA followed by a Tukey test. The AP value obtained after the injection of hexamethonium bromide was compared with the intercepts of the peripheral arcs determined under the control condition and after high-dose clonidine using one-way repeated-measures ANOVA followed by a Tukey test. The differences were considered statistically significant at *P* < 0.05.

## Results

The left panels in Fig. 1 represent the time series obtained from one rat. Under baseline conditions (S1 and S2), keeping CSP at 60 mmHg increased SNA, AP, and HR. Thereafter, the increase in CSP decreased SNA, AP, and HR. Administration of low-dose clonidine one minute after the completion of S2 (indicated by the down arrow at 2 μg/kg) decreased SNA and HR but transiently increased AP. The transient AP increase subsided by the end of S3. Although the maximum AP was lower in S4 than in S2, the minimum AP was higher in S4 than in S2. The addition of high-dose clonidine one minute after the completion of S4 (indicated by the down arrow at 5 μg/kg) further decreased SNA and HR with a transient increase in AP. The SNA was nearly silenced after high-dose clonidine. The elevated AP gradually declined toward the end of S5. The maximum AP was lower in S6 than in S2, whereas the minimum AP was higher in S6 than in S2. The right panels in Fig. 1 indicate that the remaining small burst activity of SNA (depicted in gray) disappeared after the administration of hexamethonium bromide. The top panel was virtually the same as the third panel at this time resolution because CSP was servo-controlled to mimic instantaneous AP.

**Fig. 2:**
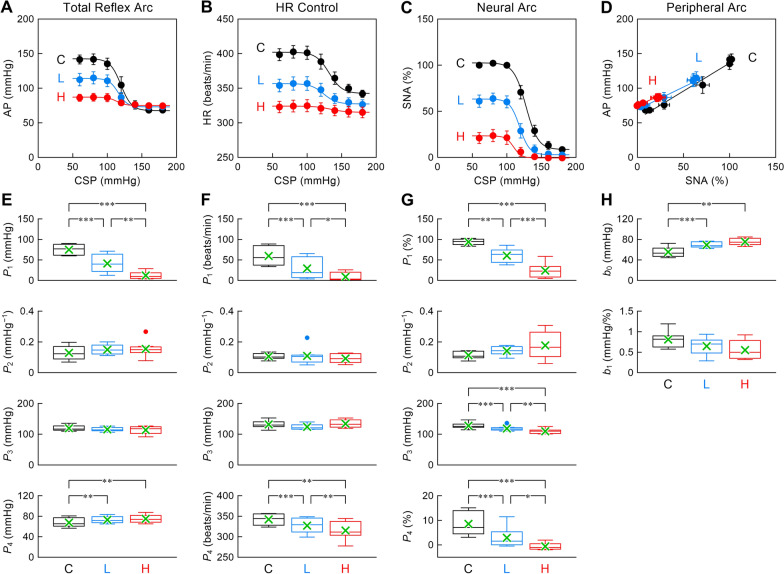
Open-loop static characteristics of the total reflex arc (**A**), HR control (**B**), neural arc (**C**), and peripheral arc (**D**) together with their parameter values (**E**–**H**). *CSP* carotid sinus pressure, *AP* arterial pressure, *HR* heart rate, *SNA* sympathetic nerve activity, *P*_*1*_ response range, *P*_2_ slope coefficient; *P*_3_ midpoint input pressure, *P*_4_ lower asymptote of the fitted sigmoid curve. Labels C, L, H denote the data under control conditions, after low-dose (2 μg/kg) clonidine, and after high-dose (5 μg/kg) clonidine, respectively. In panels **A**–**D**, data are expressed as mean ± SE values (*n* = 8). The smooth curve or the line was drawn using mean values of fitted parameters. In panels **E**–**H**, data are expressed as box and whisker plots (*n* = 8). The box indicates the 25th–75th percentile range. The horizontal line in the box indicates the median. The values above the 75th percentile plus 1.5 times the interquartile range (IQR) or those below the 25th percentile minus 1.5 times IQR are defined as outliers (filled circles). The whiskers indicate the minimum and maximum data points, excluding the outliers. In each plot, the mean of data points was superimposed in a cross symbol. *P < 0.05, **P < 0.01, and ***P < 0.001 by a Tukey test following one-way repeated-measures analysis of variance

Figure 2 illustrates the open-loop static characteristics of the total reflex arc (Fig. 2A), HR control (Fig. 2B), neural arc (Fig. 2C), and peripheral arc (Fig. 2D). The corresponding parameter values were displayed using box and whisker plots with the mean values superimposed in cross symbols (Fig. 2E–2H). The total reflex arc approximated inverse sigmoidal curves (Fig. 2A). The response range, *P*_1_, decreased as the dose of clonidine increased (Fig. 2E). No significant effect of clonidine was observed in the slope coefficient, *P*_2_, or the midpoint pressure, *P*_3_. The lower asymptote, *P*_4_, was significantly higher after clonidine administration than before. The baroreflex-mediated HR response also approximated inverse sigmoidal curves (Fig. 2B). The response range and the lower asymptote of HR decreased as the dose of clonidine increased (Fig. 2F). The neural arc approximated inverse sigmoidal curves (Fig. 2C). The response range, midpoint pressure, and lower asymptote showed a reduction dependent on the dose of clonidine (Fig. 2G). The peripheral arc approximated straight lines (Fig. 2D). The intercept, *b*_0_, was significantly higher after clonidine administration than before, whereas the slope, *b*_1_, did not differ significantly among the three conditions (Fig. 2H).

**Fig. 3:**
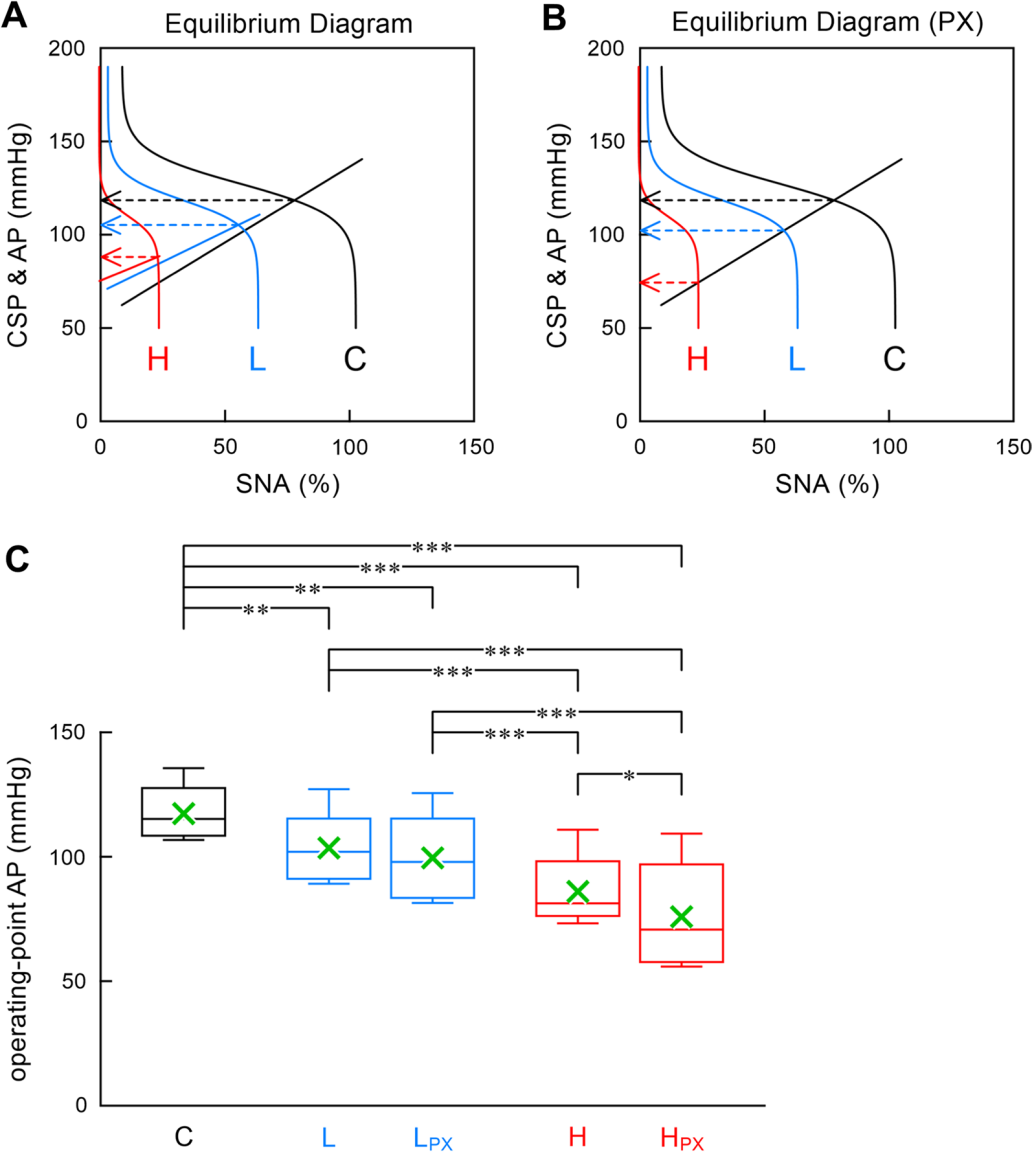
Baroreflex equilibrium diagrams constructed from fitted neural and peripheral arcs with (**A**) or without (**B**) the peripheral effect of clonidine. PX, the exclusion of the peripheral effect. Labels C, L, H denote the data under control conditions, after low-dose (2 μg/kg) clonidine, and after high-dose (5 μg/kg) clonidine, respectively. The operating-point AP was defined as the AP at the intersection point between the neural and peripheral arcs (the left arrowheads). In panel **C**, the operating-point AP values are expressed as the box and whisker plots (*n* = 8). The box indicates the 25th–75th percentile range. The horizontal line in the box indicates the median. The whiskers indicate the minimum and maximum data points. The mean of data points was superimposed in a cross symbol. *P < 0.05, **P < 0.01, and ***P < 0.001 by a Tukey test following one-way repeated-measures analysis of variance

The baroreflex equilibrium diagram was obtained by plotting the neural and peripheral arcs on a pressure–SNA plane. The operating-point AP, which was determined from the intersection point between the neural and peripheral arcs, decreased with the increasing dose of clonidine (Fig. 3A, the left arrowheads). The operating-point AP was also determined with the peripheral arc fixed at its control position, thereby excluding the peripheral effect of clonidine (Fig. 3B, the left arrowheads; PX, the exclusion of the peripheral effect). The operating-point AP after low-dose clonidine did not differ significantly with or without the peripheral effect (Fig. 3C). By contrast, the operating-point AP after high-dose clonidine was significantly higher with the peripheral effect than without.

The mean AP observed after hexamethonium was 79.3 (75.7–81.2) mmHg [median (25th–75th percentile range)]. This value remained significantly higher than the intercept of the peripheral arc estimated under the control condition [53.2 (46.8–63.2) mmHg, *P* < 0.001], whereas it was not significantly different from the intercept estimated after high-dose clonidine [74.7 (70.0–82.4) mmHg].

## Discussion

We have shown that the intravenous administration of clonidine dose-dependently suppressed SNA (Fig. 2C and 2G). Clonidine significantly increased the intercept of the peripheral arc without a statistically significant change in the slope (Fig. 2D and 2H). Although the lower asymptote of the neural arc was significantly lower after clonidine administration than before, the lower asymptote of the total reflex arc was significantly higher after clonidine administration than before (Fig. 2A and 2E).

### Effect of clonidine on baroreflex-mediated sympathetic AP regulation

Agonists for α_2_-adrenergic receptors, such as clonidine and guanfacine, are known to inhibit SNA. Studies on mice that lack specific subtypes of α_2_-adrenergic receptors indicate that the α_2A_ subtype is responsible for central sympathetic suppression [5], whereas the α_2C_ subtype contributes to peripheral vasoconstriction [15]. However, in our previous study, guanfacine, a selective α_2A_-adrenergic agonist, demonstrated a significant peripheral effect, which nearly cancels the centrally mediated reduction of the operating-point AP [7]. By contrast, the peripheral effect of clonidine did not affect the operating-point AP after the low-dose administration and only partly offset the reduction of the operating-point AP after the high-dose administration (Fig. 3C). While guanfacine and clonidine are recognized as α_2_-adrenergic agonists, α_1_-adrenergic receptors may be involved in the peripheral effect. For instance, guanfacine and clonidine exerted a vasoconstrictive effect on a rabbit aortic strip, and this vasoconstriction was prevented by prazosin, an α_1_-adrenergic antagonist [16]. In that study, the maximum contractile effect of guanfacine was close to that of norepinephrine, whereas the maximum contractile effect of clonidine was less than a half of that of norepinephrine. The pressor effect of clonidine was also observed in pithed rabbits in vivo [17], suggesting that the pressor effect is independent of ongoing SNA. In the same study, prazosin partly inhibited the pressor response to clonidine. In addition, yohimbine, an α_2_-adrenergic antagonist, blunted the pressor response to clonidine.

Moxonidine and rilmenidine are second-generation central antihypertensive agents that preferentially act on I_1_-imidazoline receptors to reduce unwanted side effects of clonidine, such as dry mouth and sedation, resulting from α_2_-adrenergic stimulation [1, 2, 4]. Nevertheless, it has been demonstrated that an expression of α_2A_-adrenergic receptors is a prerequisite for the cardiovascular effect of moxonidine and rilmenidine [18]. It is understood that I_1_-imidazoline receptors are located upstream from α_2_-adrenergic receptors in the rostral ventrolateral medulla. Activation of I_1_-imidazoline receptors increases the release of norepinephrine, which suppresses SNA by activating α_2_-adrenergic receptors [2, 19]. By contrast, sedation is mediated by α_2_-adrenergic stimulation in the locus coeruleus [20], and imidazoline receptors are only indirectly involved in the stimulation of the locus coeruleus neurons [21].

In our previous study, moxonidine exhibited a strong peripheral effect that attenuated the reduction of the operating-point AP by more than a half magnitude. While it is not classified as an α_1_-adrenergic agonist, moxonidine increases the tension of the rat tail artery in a manner dependent on α_1_-adrenergic receptors [22]. The maximum tension development was near 90% of that induced by phenylephrine, and the tension development of moxonidine was inhibited by urapidil, an α_1_-adrenergic antagonist. Clonidine also increased the tension of the rat tail artery with a maximum tension development of approximately 60% of that induced by phenylephrine [22]. After the high-dose administration, the vasoconstrictive effect clonidine attenuated the centrally mediated reduction of the operating-point AP (Fig. 3C).

Although classification based on receptor affinities helps understand the action of a given drug, it can lead to oversimplification in the interpretation of drug effects. Once a given drug is classified as a central antihypertensive, investigators are prone to attribute an observed AP change to its central effect. The present study demonstrated the importance of interpreting the AP change in terms of the relative potency of the central and peripheral effects in vivo.

### Effect of clonidine on baroreflex-mediated sympathetic HR control

In the present study, the HR response was mediated by the sympathetic system because of vagotomy. The lower asymptote of HR decreased as the dose of clonidine increased (Fig. 2F), which agrees with changes in the lower asymptote of the SNA response (Fig. 2G). Although the sympathetic HR response depends mainly on β_1_-adrenergic receptors, the sympathetic HR control can be affected by presynaptic inhibition via α_2_-adrenergic receptors. In our previous study using rabbits, clonidine infusion attenuated the release of norepinephrine during cardiac sympathetic nerve stimulation and attenuated the dynamic HR response [23]. Furthermore, clonidine exhibited an inhibitory effect on hyperpolarization-activated cyclic nucleotide-gated (HCN) channels and reduced HR in α_2ABC_-knockout mice [24]. Since the inhibition of HCN channels by ivabradine does not narrow the range of the baroreflex-mediated HR response [25], the HCN channel inhibition may not account for the reduced response range of HR after clonidine administration. On the other hand, HCN channel inhibition by clonidine may partially contribute to the reduction of the lower asymptote of HR after clonidine administration.

### Implication of the study

Sympathetic overactivity can aggravate cardiovascular diseases, such as chronic heart failure (CHF). Inhibition of peripheral sympathetic effects by β-blockers [26], angiotensin converting enzyme inhibitors [27], and angiotensin II receptor blockers [28] is widely used for the treatment of CHF. Although central sympathetic suppression may be an alternative approach for treating sympathetic overactivity, a clinical trial of moxonidine resulted in worse outcomes in CHF patients [29]. The reason for the unsuccessful treatment may be multifactorial, including the high dose of moxonidine without a slow titration period. In addition, we speculate that a peripheral effect of moxonidine might have antagonized the expected beneficial effect of afterload reduction on the failing heart [6]. In support of the interpretation, moxonidine exerted a beneficial effect in a rat model of hypertensive heart failure when administered centrally to avoid its peripheral effect [30]. Judging from the reduction of the operating-point AP (Fig. 3C), the peripheral effect of clonidine may be weaker than that of moxonidine. Although oral administration of clonidine improved survival in a rat model of CHF after myocardial infarction [31], clonidine is not ideal for the treatment of CHF because of side effects associated with α_2_-adrenergic stimulation. Further studies are required to identify central antihypertensive agents with minimal side effects.

### Limitations

First, the present results cannot account for any chronic effects of clonidine under conscious conditions because of the acute nature of the protocol. Second, there may be a species difference and a regional difference with regard to the peripheral effect relative to the central sympatholytic effect. Third, the AP response to SNA includes both the vascular and cardiac effects. Although the vascular properties play a predominant role in the baroreflex-mediated AP regulation under vagotomy [32], further studies are required to separate the vascular and cardiac effects of clonidine. Finally, we recorded splanchnic SNA as a proxy of systemic SNA. There is a possibility that sympathetic nerves other than the splanchnic sympathetic nerve responded differently to clonidine and contributed to the rise in the intercept of the peripheral arc drawn using splanchnic SNA as an abscissa. However, the total sympathetic blockade by hexamethonium at the end of the experiment did not significantly reduce AP compared with the intercept of the peripheral arc observed after high-dose clonidine. Hence, the rise in the intercept of the peripheral arc observed after high-dose clonidine likely represents a direct peripheral effect of clonidine rather than a differential regional modulation of sympathetic control.

## Conclusions

Clonidine is a first-generation central antihypertensive, and its use has declined because of side effects, such as dry mouth and sedation. However, when the baroreflex-mediated sympathetic AP regulation was in focus, clonidine represented a weak peripheral effect relative to its central sympatholytic effect. These characteristics of clonidine may serve as a reference to the understanding of other central antihypertensive agents.

## Data Availability

The datasets used and/or analyzed during the current study are available from the corresponding author on reasonable request.

## Abbreviations

ANOVA: Analysis of variance
AP: Arterial pressure
CHF: Chronic heart failure
CSP: Carotid sinus pressure
HR: Heart rate
SNA: Sympathetic nerve activity
